# Empirical validation of the wcst network structure in patients

**DOI:** 10.1192/j.eurpsy.2021.1389

**Published:** 2021-08-13

**Authors:** G. Gil-Berrozpe, A. Sánchez-Torres, L. Moreno-Izco, R. Lorente-Omeñaca, A. Ballesteros, Á.S. Rosero, V. Peralta, M. Cuesta

**Affiliations:** Mental Health Group, Instituto de Investigación Sanitaria de Navarra (IdISNA), Pamplona, Spain

**Keywords:** WCST, Empirical validation, Antecedent, concurrent and outcome validators, Network analysis

## Abstract

**Introduction:**

Cognitive impairment is a core feature of schizophrenia and other psychotic disorders and executive deficits are within the most impaired cognitive functions The Wisconsin Card Sorting test (WCST) has been extensively used in literature on schizophrenia and psychosis. The underlying structure of executive impairment may have important implications for our understanding of the complex connections between executive dysfunction and the psychopathology and neurofunctional basis of psychosis.

**Objectives:**

The objective was to empirically validate the dimensions of the WCST network structure of patients regarding antecedent, concurrent and outcome variables.

**Methods:**

Subjects were 298 patients with a DSM 5 diagnosis of psychotic disorder. To assess the empirical validation of network structure of the WCST antecedent, concurrent and outcome variables were selected from the CASH interview and other scales of patients.

**Results:**

Pearson coefficient correlations between the 4 network loadings (NL) of WCST, namely perseveration, inefficient sorting, failure to maintain the set and learning, and antecedent, concurrent and outcome validators are shown in the table. PER and IS showed common and strong associations with antecedent, concurrent and outcome validators. LNG dimension was also moderately associated and FMS did not show significant associations.

**Conclusions:**

‘Perseveration’ and ‘Inefficient sorting’ dimensions achieve and share common antecedent, concurrent and outcome validators. While ‘Learning’ dimension achieves partial validation in terms of antecedent and outcome validators and ‘Failure to maintain the set’ dimension was not associated with external validators. These four underlying dysfunctions might help to disentangle the neurofunctional basis of executive deficits in psychosis.

